# Reliable and Efficient Approach of BOLD Signal with Dual Kalman Filtering

**DOI:** 10.1155/2012/961967

**Published:** 2012-09-10

**Authors:** Cong Liu, Zhenghui Hu

**Affiliations:** State Key Laboratory of Modern Optical Instrumentation, Zhejiang University, Hangzhou 310027, China

## Abstract

By introducing the conflicting effects of dynamic changes in blood flow, volume, and blood oxygenation, Balloon model provides a biomechanical compelling interpretation of the BOLD signal. In order to obtain optimal estimates for both the states and parameters involved in this model, a joint filtering (estimate) method has been widely used. However, it is flawed in several aspects (i) Correlation or interaction between the states and parameters is incorporated despite its nonexistence in biophysical reality. (ii) A joint representation for states and parameters necessarily means the large dimension of state space and will in turn lead to huge numerical cost in implementation. Given this knowledge, a dual filtering approach is proposed and demonstrated in this paper as a highly competent alternative, which can not only provide more reliable estimates, but also in a more efficient way. The two approaches in our discussion will be based on unscented Kalman filter, which has become the algorithm of choice in numerous nonlinear estimation and machine learning applications.

## 1. Introduction

A thorough understanding of the dynamic relationship between cerebral blood flow (CBF), cerebral blood volume (CBV), and the blood oxygenation level dependent (BOLD) signal is essential for the physiological interpretation of fMRI activation data. The Balloon model described by Buxton et al. (1998) [1] is the first biomechanical plausible model to expound this relationship: increasing the flow (or perfusion rate) generally leads to dilution of venous deoxyhemoglobin (dHb), reducing the tendency of the blood to attenuate the magnetic resonance signal. The resultant increase in signal intensity is referred to as the BOLD response [2]. It is by extending this model to cover the dynamic coupling between CBF and synaptic activity, more sophisticated physiological realities are incorporated, for example, oxygen metabolism dynamics, both intra- and extravascular signal [3, 4], and more intricate models obtained.

The Balloon model is an input-state-output model with three state variables: blood flow, and volume, deoxyhemoglobin content and several biologically reasonable parameters. The problems of state estimation and parameter estimation (sometimes referred to as system identification or machine learning) associated with Balloon model are often formulated in a state-space representation, where Balloon model serves as a set of continuous-time system equations to describe the hemodynamic process. The equations are nonlinear, corresponding to the fact that Balloon model is one of the numerous nonlinear approaches to characterizing evoked hemodynamic response in fMRI.

Several work has utilized the Balloon model or its enhanced versions in the analysis of fMRI response. Some approaches, including expectation maximization (EM) [5, 6] and maximum likelihood [7], model the BOLD observation as deterministic hemodynamic process. However, a limitation of these methods is that they can only deal with measurement noise. Many promising approaches to the dual estimation problem belong to filtering algorithm that is able to account for both the physiological and measurement noise. Riera et al. [8] addressed the data assimilation problem in an extended Kalman filter (EKF) strategy. As EKF might lead to the problem of divergence due to linearized approximation, Johnston et al. proposed particle filter [9, 10] to avoid the flaw of linearization. Moreover, Hu et al. [11–13] employed unscented Kalman filter (UKF) that also outperforms EKF in terms of estimation error but with roughly the same computational cost. Most recently, Friston et al. described variational filtering to optimize the approximation of posterior density on hidden model variables, while accumulating sufficient statistics to optimize the conditional densities of parameters and precision [14, 15].

The approaches mentioned above have greatly improved our ability to explore, and above all, to quantify the physiological mechanism involved in neural activation. However, they still have palpable defects. It is noteworthy that many of them actually are in the spirit of joint filtering, in which the underlying states and parameters are concatenated into a single higher dimensional joint state space, a filter runs for estimating both the states and parameters. Despite its straightforwardness in theory and convenience in implementation, the weakness of joint filtering is obvious. The objective of this paper is to introduce and develop an estimator equally concise but with higher performance—dual filtering. We will demonstrate the advantage of dual filter from two aspects, by the example of dual UKF versus joint UKF. (1) In terms of Balloon model, there is no inherent biophysical correlation between the states and parameters. By treating them separately, dual filtering can avoid undesired transaction between them. (2) Larger dimension of state-space vector implies much more computational expense. Specifically, computational complexity for general state-space problems is *𝒪*(*L* ^3^) [16]. Although the frequency of predict-update cycle required by dual filter is the twice of that required by joint filter, dual estimate is much more computational efficient.

## 2. Materials and Methods

### 2.1. Hemodynamic Model

 Balloon model describes the coupled kinetic changes from synaptic activity to the fMRI BOLD signal at a given region. This model has been extended by Friston et al. (2000) [5] to include the effects of external inputs to an autoregulated vasodilatory signal, assuming that the relationship between evoked neural activity and blood flow is linear. The subsequent work added different variations to this model, several of them were reviewed and integrated in Stephan et al. (2004) [18] and Buxton (2004) [19]. Based on fundamental physiology, rather than empirical approaches, these enhanced models are able to unify existing literature and provide insight into how the underlying physiological mechanisms result in stable or/and transit BOLD response. However, the original model proposed by Buxton et al. and completed by Friston et al. is sufficient to account for the nonlinear behaviors observed in real-time series [5]. Too many state variables and parameters will not serve our purpose here better.

The dynamic intertwinement between multiple physiological variables, the cerebral blood flow (CBF) *f*, blood venous volume *v*, and veins deoxyghemoglobin content *q*, can be given as a set of nonlinear nondimensional differential equations [1, 20]:

(1) $$\begin{array}{c} \ddot{f}=\epsilon u\left(t\right)-\frac{\dot{f}}{\tau_{s}}-\frac{f-1}{\tau_{f}},\\ \dot{v}=\frac{1}{\tau_{0}}\left(f-v^{1/\alpha }\right),\\ \dot{q}=\frac{1}{\tau_{0}}\left(f\frac{1-{\left(1-E_{0}\right)}^{1/f}}{E_{0}}-v^{1/\alpha }\frac{q}{v}\right), \end{array}$$

where *ϵ* is neuronal efficacy, reflecting the significance of neuronal activity evoked by experimental event, hence it varies with trial event; *τ*_*s*_ and *τ*_*f*_ represent time constant for signal decay and autoregulatory feedback from blood flow, respectively. The existence of feedback term can be inferred from the poststimulus undershoots in CBF [21]. The degree of nonlinearity of the BOLD signal is largely determined by the stiffness parameter *α*, which characterizes the balloon-like capacity of the venous compartment to expel blood at a greater rate when distended [22]. *E*_0_ is resting net oxygen extraction fraction. All variables are expressed in normalized form, relative to resting values.

Noticing that the first equation has a second-order time derivative, so we can write this input-state-output system as a set of first-order ordinary differential equations by introducing another variable $s=\dot{f}$. By defining the state vector as **x**(*t*) = [*f*, *s*, *q*, *v*]^*T*^, the system dynamic equation can be constructed from (1):

(2) $$\begin{array}{c} \dot{x}=f\left(x,\theta ,u,v\right)\;v\sim N\left(0,Q\right), \end{array}$$

where *θ* = {*ϵ*, *τ*_*s*_, *τ*_*f*_, *τ*_0_, *α*, *E*_0_, *V*_0_} ∈ ℝ^*l*^ is system parameter, the neuronal input **u** represents system input, and **v** is to account for the process noise.

The observed signal can be taken as a nonlinear function of volume *v* and deoxyghemoglobin *q* that comprises a volume-weighted sum of intra- and extravascular signal:

(3) y(t)=V0(k1(1−q)+k2(1−qv)+k3(1−v)),k1=7E0,    k2=2,    k3=2E0−0.2,

appropriate for a 1.5 tesla magnet [1]. *V*_0_ is the resting blood volume fraction, which generally varies across brain regions and subjects. All parameters are independent of each other. Their physiological definitions and probability distributions are given in Table 1 [23].

The actual observation **y** is then composed of a deterministic part *h*(*x*, *θ*, *t*) and a stochastic part **w**:

(4) $$\begin{array}{c} y=h\left(x,\beta ,w\right)\;w\sim N\left(0,R\right), \end{array}$$

where **y** is the observation vector, **w** is measurement noise, and *β* consists of *k*_1_, *k*_2_, and *k*_3_. Simultaneous estimation of *V*_0_ and other parameters would be impossible, since their product is settled for each sampled measurement *y*_*k*_. The stiffness parameter *α* is a nominal factor to BOLD contrast, it can be fixed to any value with its reasonable range in system identification [17].

Equation (2) describes a continuous-time hemodynamic process, and (4) models fMRI measurement as discrete sampling of the continuous system states, together they have formed a standard state-space representation for fMRI data assimilation. Given *y*_*k*_, the physiological states **x** and the optimal parameters for a certain voxel can be estimated by use of UKF—a recursive minimum mean-square-error (MMSE) estimator.

### 2.2. Dual UKF and Joint UKF

 The unscented Kalman filter has been applied extensively to the field of nonlinear estimation for both states and parameters. The basic framework of UKF involves estimation of the states of a discrete-time nonlinear dynamic system:

(5) $$\begin{array}{c} x_{k+1}=F\left(x_{k},u_{k},v_{k}\right),\\ y_{k}=H\left(x_{k},n_{k}\right), \end{array}$$

where **x**_*k*_ represents the unknown system states, the system is driven by a known exogenous input **u**_*k*_ and process noise **v**_*k*_. The observation noise is given by **n**_*k*_.

The UKF generally involves recursive utilization of a deterministic “sampling” approach. The sampled points (sigma points) completely capture the true mean and covariance of the variables, and when propagated through the nonlinear system (**F** in this case), they are able to capture the posterior mean and covariance accurately to the 2nd order of Taylor series expansion [16].

Parameter estimation, or machine learning, on the other hand, involves determining a nonlinear mapping:

(6) $$\begin{array}{c} y_{k}=G\left(x_{k},w\right), \end{array}$$

where **x**_*k*_ is the input, **y**_*k*_ is the output, and the nonlinear map **G**(·) is parameterized by the vector **w**. Typically, a training set is provided by sample pairs consisting of known input and desired output, {**x**_*k*_, **d**_*k*_}. The goal of the learning can be expressed to some degree as solving for **w** which minimizes the error of the machine: **e**_*k*_ = **d**_*k*_ − **G**(**x**_*k*_, **w**). In order to estimate the parameters by utilizing UKF, a new state-space representation can be written:

(7) $$\begin{array}{c} w_{k+1}=w_{k}+r_{k},\\ d_{k}=G\left(x_{k},w_{k}\right)+e_{k}, \end{array}$$

where the parameters **w**_*k*_ correspond to a stationary process with identity state transition matrix, driven by process noise **r**_*k*_.

Given that BOLD signal is the only output and observation of the system in terms of Balloon model, the dual estimation problem, in which the system states and model parameters must be estimated simultaneously, can be given as follows:

(8) $$\begin{array}{c} x_{k+1}=F\left(x_{k},u_{k},v_{k},w_{k}\right),\\ w_{k+1}=w_{k}+r_{k},\\ y_{k}\;=G\left(x_{k},w_{k}\right)+e_{k}. \end{array}$$

Since standard UKF cannot be applied to this system immediately, dual UKF and joint UKF have been proposed as two alternatives. In the dual filtering method, two UKFs—one for state estimation, the other for parameter estimation—run in an alternate way. At each time step, the current estimate of parameters ${\hat{w}}_{k}$ is used in the state filter as given input, and likewise the current states estimate ${\hat{x}}_{k}$ is used in the parameter filter. On the contrary, a single UKF runs for both state and parameter estimation in the joint filtering. A higher dimensional joint state vector is defined: ${\tilde{x}}_{k}={\left[{x_{k}}^{T}{w_{k}}^{T}\right]}^{T}$, and the state-space model is reformed as follows:

(9) $$\begin{array}{c} {\hat{x}}_{k+1}=\hat{F}\left({\hat{x}}_{k},u_{k},{\hat{v}}_{k}\right),\\ y_{k}=G\left({\hat{x}}_{k},n_{k}\right). \end{array}$$

**Figure 1:**
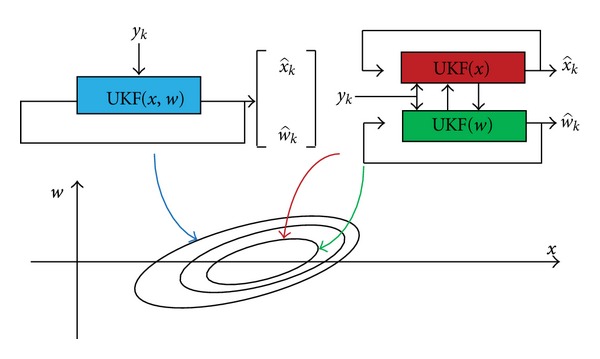
Schematic diagrams of joint filter (left) and dual filter (right).

The dual UKF and joint UKF approaches are illustrated in Figure 1.

In this section, the framework of UKF is briefly reviewed, a dual estimation problem with two approaches have been presented. In the next section, we will focus on examining the different performances of dual UKF and joint UKF, and all of our discussion will be in the context of Balloon model.

## 3. Results and Discussion

### 3.1. Biophysical Interpretation

 One of the most prominent bifurcations between dual estimate and joint estimate is whether to incorporate interaction or correlation between states and parameters into filtering. As discussed earlier, the joint filter concatenates the state and parameter random variables into a single augmented state (${\hat{\tilde{x}}}_{k}$), so the cross-covariance between states and parameters is effectively modeled, that is,

(10) $$\begin{array}{c} E\left[\left(\hat{\tilde{x}}-E\left[\hat{\tilde{x}}\right]\right){\left(\hat{\tilde{x}}-E\left[\hat{\tilde{x}}\right]\right)}^{T}\right]=\left[\begin{array}{cc} P_{x_{k}x_{k}} & P_{x_{k}w_{k}}\\ P_{w_{k}x_{k}} & P_{w_{k}w_{k}} \end{array}\right]. \end{array}$$

Dual filtering, on the other hand, decouples (in a statistical sense) the dual estimation problem by treating states and parameters separately, which means **P**_*x*_*k*_*w*_*k*__ = **P**_*w*_*k*_*x*_*k*__ = 0. For states and parameters involved in Balloon model, no dynamic interaction or biophysical correlation between them has been observed (they are uncorrelated variables), therefore, it is reasonable to expect dual filtering to exhibit more biophysical accuracy. Thus the fMRI experiments substantiated our assumption.

**Figure 2:**
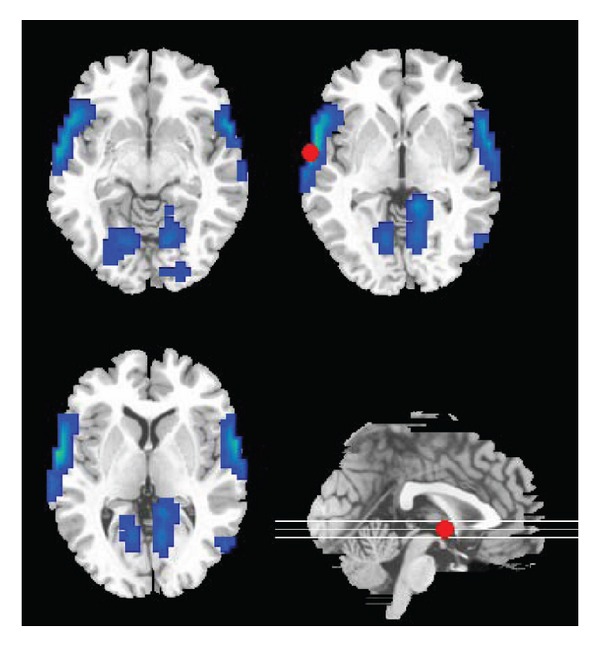
The greatest activated area of the group in the superior temporal gyrus (GT) for data assimilation.

The real fMRI data was acquired from 8 health subjects. 136 acquisitions in total were made (RT = 2s), in block of 8, giving 16 16-second blocks. The condition for successive blocks alternated between rest and auditory stimulation, starting with rest. Auditory stimulation was emotionally neutral words presented at a rate of 60 per minute. We selected the largest activated voxels in superior temporal gyrus (GT) to implement data assimilation [24] (Figure 2). Bias correction was performed using the method in [25]. The two algorithms were initialized in identical way on experimental data and parameters.

**Figure 3:**
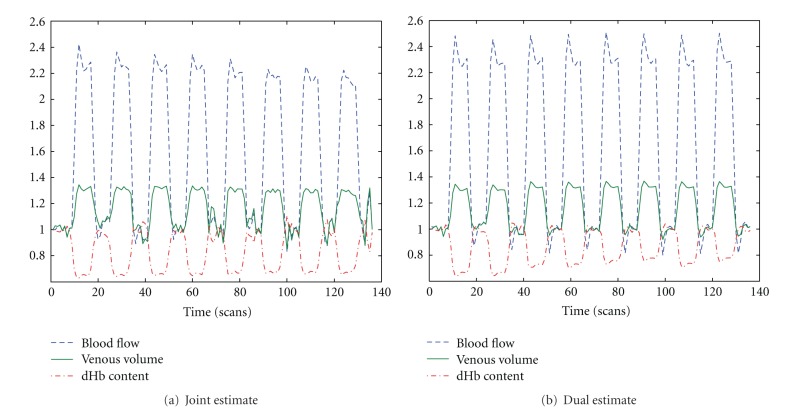
States estimated by dual and joint UKF. The dotted line corresponds to change of blood flow, the solid line shows venous volume and dashed line depicts dHb content.

**Figure 4:**
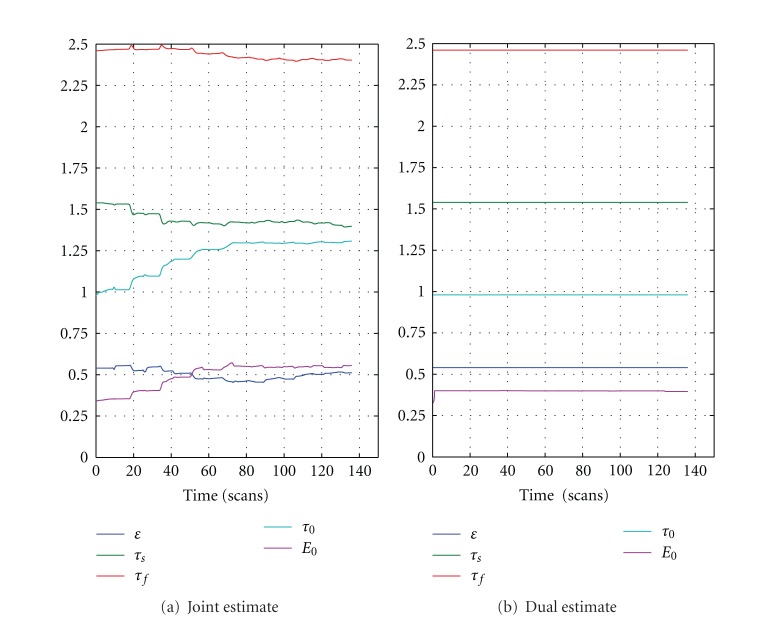
Parameters estimated by joint UKF and dual UKF. The mean values in Table 1 are used as initial values in our simulation. Apart from *E*_0_, which grows sharply at the beginning and does not change afterwards, all the parameters obtained from dual UKF can be seen as constants. Resting oxygen extraction *V*_0_ is the most important parameter in driving the model uncertainty [17], but simultaneous estimation of *V*_0_ and other parameters would be impossible, as stated earlier. *α* is a nominal mechanism to BOLD signal. Therefore *V*_0_ and *α* does not enter filtering.

Figure 3 shows the hemodynamic states given by joint UKF and dual UKF. The lower peak of blood flow inferred from joint UKF corresponds to the smaller neuronal efficacy (*ϵ*) in Figure 4.

Parameters estimated are shown in Figure 4. Signal decay, autoregulation et al. remain unchanged (almost) during dual filtering. While for joint UKF, the parameters do not converge to their final values until the 4th ~ 5th block (60 ~ 80 s after the first stimulation). This phenomenon is a strong indicator for the introduced interaction between states and parameters.

**Figure 5:**
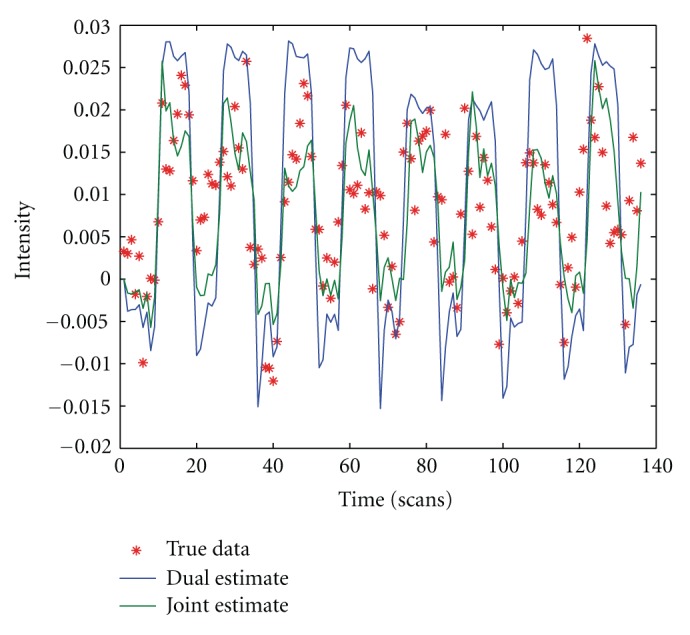
Estimated and real fMRI signal.

Real and estimated fMRI signals are plotted in Figure 5. The simulated BOLD signal given by dual UKF shows a slight overshoot, followed by gradual return to reduced plateau, and ending with a strong poststimulus undershoot. On the other hand, joint UKF fails to some degree to reconstruct a clean BOLD signal: the plateau is missing, neither does the evolving pattern of the signal show itself in a stable way in each block. The overestimated transit time (*τ*_0_) leads to a reduction in amplitude of the BOLD peak; the less intense poststimulus undershoot can be explained by the underestimated signal decay (*τ*_*s*_). Given the fact that dual UKF and joint UKF have very similar performance for state estimation, which is made clear in Figure 3, it is safe to attribute the failure to the undesired fluctuations of parameters (especially *E*_0_, which affects the estimated signal directly by (3)), or more precisely, the undesired interaction between parameters and states.

### 3.2. Computational Interpretation

 One of the most computationally expensive operations in UKF corresponds to calculating the new set of sigma points at each time update. This requires taking a matrix squareroot of the state covariance matrix, **P** ∈ ℝ^*L*×*L*^, given by **S** **S** ^*T*^ = **P** [16]. An efficient implementation using a Cholesky factorization requires in general *𝒪*(*L* ^3^/6) computations [26]. Therefore enlarging the dimension of state-space vector will dramatically increase the computational complexity (also can be referred to as time complexity). In this subsection we will introduce two criteria for evaluating the property of dual UKF and joint UKF in time complexity.

*Number of Floating-Point Operations* (*flops*). In computing, floating point can be thought of as a computer realization of scientific notation, which is able to represent a wide range of values. Flops number required by a given algorithm or computer program is independent of the computing platform, although its precise value may differ under different counting rules. MATLAB (version before 6.0) has provided us with a useful function *flops* to specify the cumulative number of flops. For instance, if *A* and *B* are *N*-by-*N* matrixes, then the output of *flops*  (*A* + *B*) and *flops*  (*AB*) will approximately be *N* ^2^ and 2*N* ^3^.

Since Cholesky factorization is the only operation within UKF whose time complexity is proportional to *N* ^3^, it is appropriate to consider that the flops number of Cholesky factorization is sufficient to determine the flops of the whole algorithm. For Balloon model, the dual estimation problem is about determining four state variables (*L*_*x*_ = 4) and five parameters (all the parameters except *V*_0_ and *α*, *L*_*w*_ = 5) at each time step.  Table 2 shows the total flops number of Cholesky factorization involved in each predict-update cycle of UKF.

However, in practice by slightly restructuring the state vector, the process and observation models, we may introduce the noise with the same order of accuracy as the uncertainty in the state. First, the state vector is augmented to give a *L* ^*α*^ = *L*_*x*_ + *L*_*v*_ + *L*_*n*_ dimensional vector;

(11) $$\begin{array}{c} x_{k}^{\alpha }=\left[\begin{array}{c} x_{k}\\ v_{k}\\ n_{k} \end{array}\right]. \end{array}$$

Then the process model is rewritten as a function of **x**_*k*_^*α*^, **x**_*k*+1_^*α*^ = **F**(**x**_*k*_^*α*^, **u**_*k*_); the unscented transform uses sigma points that are drawn from

(12) $$\begin{array}{c} P^{\alpha }=\left[\begin{array}{ccc} P_{x} & 0 & 0\\ 0 & R_{v} & 0\\ 0 & 0 & R_{n} \end{array}\right], \end{array}$$

where **R**_*v*_ and **R**_*n*_ are the process and observation noise covariance. In this situation, similarly we can derive Table 3.

For either case mentioned above, the flops number for joint filtering is at least 56% larger than that for dual filtering.

Comparing to flops count, overall execution time is a more tangible and practical criterion. We have tested our programs on several computers and collected their execution time data. Normally, a difference over 90% can be observed (16 s and 30 s for dual UKF and joint UKF, resp.). This result is even more impressive than that related to flops analysis, indicating that flops number is not the only factor influencing the operation time. Even if we take the rapid improvements in processing speed and memory into consideration, this variance is significant and should not be ignored.

## 4. Conclusions

 In this paper we brought forward the dual Kalman filter as a reliable and efficient approach to estimating the states and parameters involved in balloon model. Comparing to the commonly used joint Kalman filter, its principle is in better conformity with the physiological reality, and by decoupling the dual estimation problem, it is much more calculational efficient to implement. The result of experiments showed good agreement with our conclusion.

## Figures and Tables

**Table 1:** Hemodynamic model parameters and their probability distribution.

Notation	Definition	Distribution
*ϵ*	Neuronal efficacy	*ϵ* ~ *N*(0.54,0.1^2^)
*τ*_*s*_	Signal decay	*τ*_*s*_ ~ *N*(1.54,0.25^2^)
*τ*_*f*_	Autoregulation	*τ*_*f*_ ~ *N*(2.46,0.25^2^)
*τ*_0_	Transit time	*τ*_0_ ~ *N*(0.98,0.25^2^)
*α*	Stiffness parameter	*α* ~ *N*(0.33,0.045^2^)
*E*_0_	Resting oxygen extraction	*E*_0_ ~ *N*(0.34,0.1^2^)
*V*_0_	Resting blood volume fraction	*V*_0_ ~ *N*(0.02,0.005^2^)

**Table 2:** The fact that joint filtering requires half of the iterations that are required by dual filtering has been taken into account.

Algorithm	Dimension of state vector	Total flops
Joint UKF	*L* = *L*_*x*_ + *L*_*w*_ = 9	285
Dual UKF	*L*_*x*_ = 4, *L*_*w*_ = 5	170

**Table 3:** Flops number for augmented state vector.

Algorithm	Dimension of state vector	Total flops
Joint UKF	*L* = *L*_*x*_ + *L*_*w*_ = 19	2470
Dual UKF	*L*_*x*_ = 9, *L*_*w*_ = 11	1582
